# IonCRAM: a reference-based compression tool for ion torrent sequence files

**DOI:** 10.1186/s12859-020-03726-9

**Published:** 2020-09-09

**Authors:** Moustafa Shokrof, Mohamed Abouelhoda

**Affiliations:** 1 Faculty of Computer Science, University of California at Davis, Davis, CA, USA; 2 King Faisal Specialist Hospital and Research Center, Riyadh, Saudi Arabia; 3 Saudi Human Genome Program, King Abdulaziz City for Science and Technology (KACST), Riyadh, Saudi Arabia; 4 Systems and Biomedical Engineering Department, Faculty of Engineering, Cairo University, University Square, Giza, Egypt

## Abstract

**Background:**

Ion Torrent is one of the major next generation sequencing (NGS) technologies and it is frequently used in medical research and diagnosis. The built-in software for the Ion Torrent sequencing machines delivers the sequencing results in the BAM format. In addition to the usual SAM/BAM fields, the Ion Torrent BAM file includes technology-specific *flow signal* data. The flow signals occupy a big portion of the BAM file (about 75% for the human genome). Compressing SAM/BAM into CRAM format significantly reduces the space needed to store the NGS results. However, the tools for generating the CRAM formats are not designed to handle the flow signals. This missing feature has motivated us to develop a new program to improve the compression of the Ion Torrent files for long term archiving.

**Results:**

In this paper, we present IonCRAM, the first reference-based compression tool to compress Ion Torrent BAM files for long term archiving. For the BAM files, IonCRAM could achieve a space saving of about 43%. This space saving is superior to what achieved with the CRAM format by about 8–9%.

**Conclusions:**

Reducing the space consumption of NGS data reduces the cost of storage and data transfer. Therefore, developing efficient compression software for clinical NGS data goes beyond the computational interest; as it ultimately contributes to the overall cost reduction of the clinical test. The space saving achieved by our tool is a practical step in this direction. The tool is open source and available at Code Ocean, github, and http://ioncram.saudigenomeproject.com.

## Background

Ion Torrent is one of the widely used Next Generation Sequencing (NGS) technologies, with a market share of 20% (Research and Market Report 2016). This technology is particularly popular in the medical domain, because it is fast and cost effective. It is basically used for clinical gene panels and whole exome sequencing. Gene panels are used to read the sequences of selected genes to screen for variations related to some inherited disorders [1–5] and cancer [6, 7]. Whole exome sequencing covers the whole set of genes and is mostly used to identify novel mutations and genes [8–12]. The Ion Torrent technology is not favored for whole genome sequencing due to its limited throughput, which would lead to insufficient depth for clinical use.

For clinical labs, the NGS data should be retained for a certain period of time [13]. Accreditation entities, such as the College of American Pathologists, require that the NGS laboratory maintains the data (as it is) for at least two years (2017 CAP Regulation MOL.35870, revised 08/17/2016). This requirement necessitates that the NGS lab possesses a high capacity storage systems either in site or in the cloud. For either option, the cost of data storage is part of the total cost for provisioning the service per sample. Therefore, efficient data compression should be implemented to reduce the storage footprint, which in turn reduces the cost of the test.

For medical applications, the NGS analytical pipeline starts with the step of base calling, where the physical signals (either images or electrical signals) are translated to sequences of nucleotide bases. The output of this step is a sequence file composed of a set of reads in the fastq format as in Illumina technology or in the unaligned BAM format as in Ion Torrent technology. (The read is the sequence of a DNA fragment). The BAM file is the binary version of the readable SAM text file [14]. The fastq/SAM/BAM format includes the NGS reads and related quality scores [14]. The next step of the pipeline is to align the NGS reads to the reference human genome. The output of this step is a file in the SAM/BAM format with alignment information. Ion Torrent machines have a built in software for base calling and alignment, called Torrent Suite (https://github.com/iontorrent/TS). The Torrent Suite delivers the sequencing results in *unaligned* BAM format. If the user runs the alignment and variant calling workflow, then the reads are aligned to the reference human genome and the results are kept in an *aligned* BAM format. The unaligned reads are kept as well in the aligned BAM file but without mapping information. The unaligned BAM file is deleted after the successful generation of the BAM file. In the following parts of the paper, we will use the word “BAM” to simply refer to the “aligned BAM”. The final step of the analysis pipeline is the variant calling step to identify variants (mutations) compared to the reference human genome. The challenge in this step is to discriminate genuine variants from sequencing errors. The output of this step is tabular file (VCF format) including list of mutations.

The NGS data access cycle is composed of three main phases:

1. *Analysis*, where the NGS files are accessed to run the alignment and variant calling steps of the variant analysis workflow. This phase requires direct access to the reads from a fast storage at very high IO speed. It is preferred to run this step on SSD based storage [15].
2. *Interpretation*, where the clinical experts sometimes access the BAM/CRAM files to visualize and review the alignment at certain positions. This phase does not involve computation, and it is fine that the data moves to moderate speed storage (hard-disk based). The interpretation phase terminates by issuing a clinical report to the patient with the findings and the case is then considered closed.
3. *Long term archiving*, where the data can move to high capacity slow storage (disk based or tapes) and kept inert, unless needed.

The BAM file is the largest output of this step and this is the one that should be the main target of compression. For a whole exome sequencing in clinical setting, the BAM file is in the range of 30–50 GB. The Gene Panel file is in the range of 1G–10G, but usually one runs multiple samples in the same run. The VCF files are relatively small and they are in the range of a few Megabytes. Optimizing the cost of the storage is critical for the third phase including long term archiving, where the data is kept inert for long time and is only decompressed if needed.

Different compression tools have been developed to compress the SAM/BAM files. The recent survey papers [16–18] include a description and comparison of these software tools. Broadly, these tools can be categorized into two big groups: 1) Non-reference based compression and 2) Reference based compression. Non-reference based methods compress the data by making use of its intrinsic characteristics. Reference-based methods work as follows: They first align the reads to a reference sequence. Then they compress the alignment information, which is enough to decompress the reads given the reference sequence. The reference based methods achieve high compression ratio, because the reads are almost identical to the reference except for few individual variations and sequencing errors. Reference and non-reference based compression tools can have a lossy and lossless version. For medical applications, only the lossless version should be used.

For medical applications, where the human genome (hg19 or GRC38) is used as a reference, the reference-based compression would be the method of choice for compressing the NGS data. Fritz et al. have introduced the CRAM format and related reference based compression tool [19]. Shortly after its introduction, the CRAM format became so popular that it is currently accepted by the major public NGS repositories, such as NCBI and ENA. The CRAM related method has been implemented in different tools, such as CRAMtools (www.ebi.ac.uk/ena/software/cram-toolkit), SAMtools [14] (https://github.com/samtools), Picard (http://picard.sourceforge.net), and Scramble [20]. These tools can produce output in CRAM format from SAM or BAM files.

In addition to its usual fields, the Ion Torrent BAM file includes flow signal data for each read. The flow signal is a vector of numerical integer values ∈ Z, usually bounded in practice. The flow signal vectors represent the measurements corresponding to the change in pH during base hybridization. The flow signal data cannot be discarded because it is used by the Torrent Suite to improve the accuracy of the variant calling.

As we will demonstrate in the experiment section, the flow signals occupy about 75% of the BAM file size for the human genome. Converting the BAM files to CRAM files lead to about 35% reduction in the file size. By examining the CRAM files, we figured out that the flow signals occupy about 77% of the file size. This shows that there a room for improvement and extra compression can be achieved by targeting the flow signals with a special compression procedure.

In this paper, we present the IonCRAM program to compress the Ion Torrent BAM files for long term archiving. It is *lossless reference-based compression* tool aiming at improving the space saving compared to the BAM and CRAM formats.

As we will show in the experimental results, IonCRAM could achieve an average space saving of about 43% compared to the BAM file. Compared to the CRAM format, IonCRAM achieves an extra space saving of about 8–9%.

## Implementation

### The flow signals

In this section, we provide information about the flow signals and explain how they are generated and stored in the BAM file. Ion Torrent is a Next Generation Sequencing technology based on the use of CMOS semiconductor chips, where the DNA bases are determined by sensing the release of hydrogen atoms during the hybridization process [21, 22]. The details are as follows: The DNA molecule is first fragmented into short fragments, usually around 500–1000 bps. Each single-stranded fragment is attached to a bead (a particle called ion sphere), where it undergoes a reaction to produce multiple copies of the same fragment. These copies are referred to as the *template*. The beads are then moved to the sequencing CMOS chip. The chip is composed of millions of wells and each well includes a sensor to detect the change in pH. Ideally, each ion sphere should reside in one well in the sequencing ship.

**Fig. 1:**
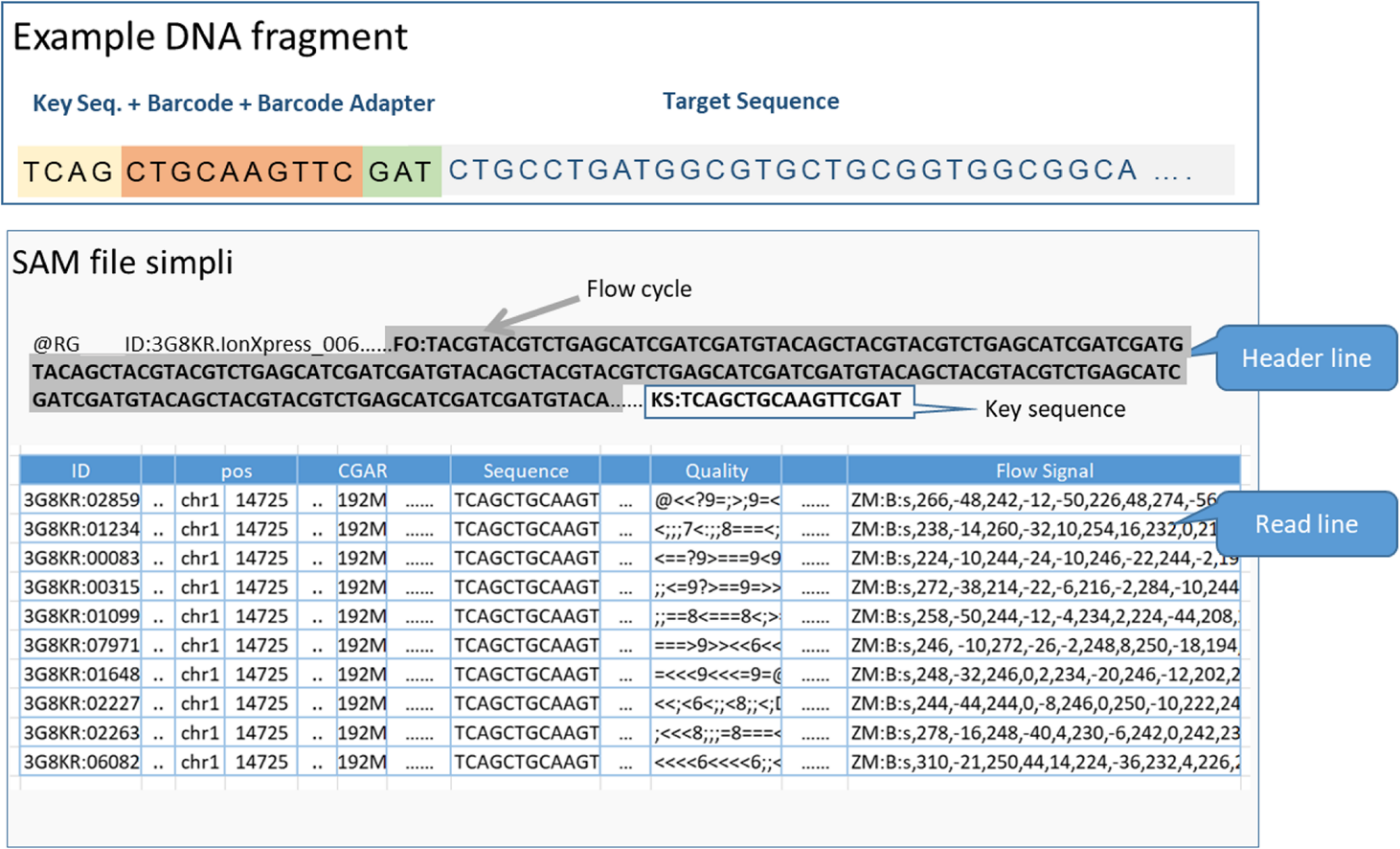
Flow Signals and their position in the SAM/BAM file. The upper part shows an example DNA fragment to be sequenced by an Ion Torrent machine. The key and bar code sequences are ligated (pre-pended) to the fragment. The key sequence (TCAG) is a control sequence to ensure correct sequencing. A barcode sequence is added to a certain group of fragments. The use of barcodes makes it possible to sequence the DNA of different samples/patients in one run. The lower part of the figure shows a schematic representation of the fields in the SAM file. The SAM file is the non-binary readable version of the BAM. The header part includes the flow cycle and the key sequence. Each line in the SAM file represents one read, aligned to the reference genome. The remaining rows include the read information in a tab-separated format: We show only the columns/fields of relevance to this paper. We show the fields including the read ID, the physical position and the CGAR string which represents the alignment, the bases of the DNA sequence in the read, the quality field, and the flow signals in the ZM field

The chip is then placed in the sequencer and the sequencing process proceeds as follows: The sequencer introduces the four bases (A, T, G, and C) one at a time during the run in a cyclic fashion. The order in which the nucleotides are introduced is referred to as *flow cycle*. An introduced nucleotide hybridizes to the template base if it is complementary to it, and a change in pH takes place. If the template at one site includes a polymer (e.g., AAA), then multiple bases can hybridize in the same round of the cycle, and this leads to a stronger change in pH. If no change is measured in one round of the cycle, then the base in the template does not match the one in the flow cycle and no hybridization reaction takes place. A wash step occurs after the introduction of each type of nucleotide to ensure no nucleotide remains in the well before the introduction of the next one in the flow cycle. The changes in pH at each round in the flow cycle are recorded, and a vector called the *raw flow-signal* is produced. The signal processing software analyzes the raw flow signals and produces a vector of processed flow signals that are eventually stored in the BAM file [21, 22]. The flow signals are numerical integer values, usually bounded in practice. The number of flow signal points is the same as the number of bases in the flow cycle. Figure 1 shows an example DNA fragment and shows how the information related to the flow cycle and the flow signals are stored in the SAM/BAM file. The string defining the flow cycle is stored once in the header of the BAM file. As also shown in the figure, each read includes information related to the quality and alignment. It also includes the flow signal vector in the “ZM” field.

**Fig. 2:**
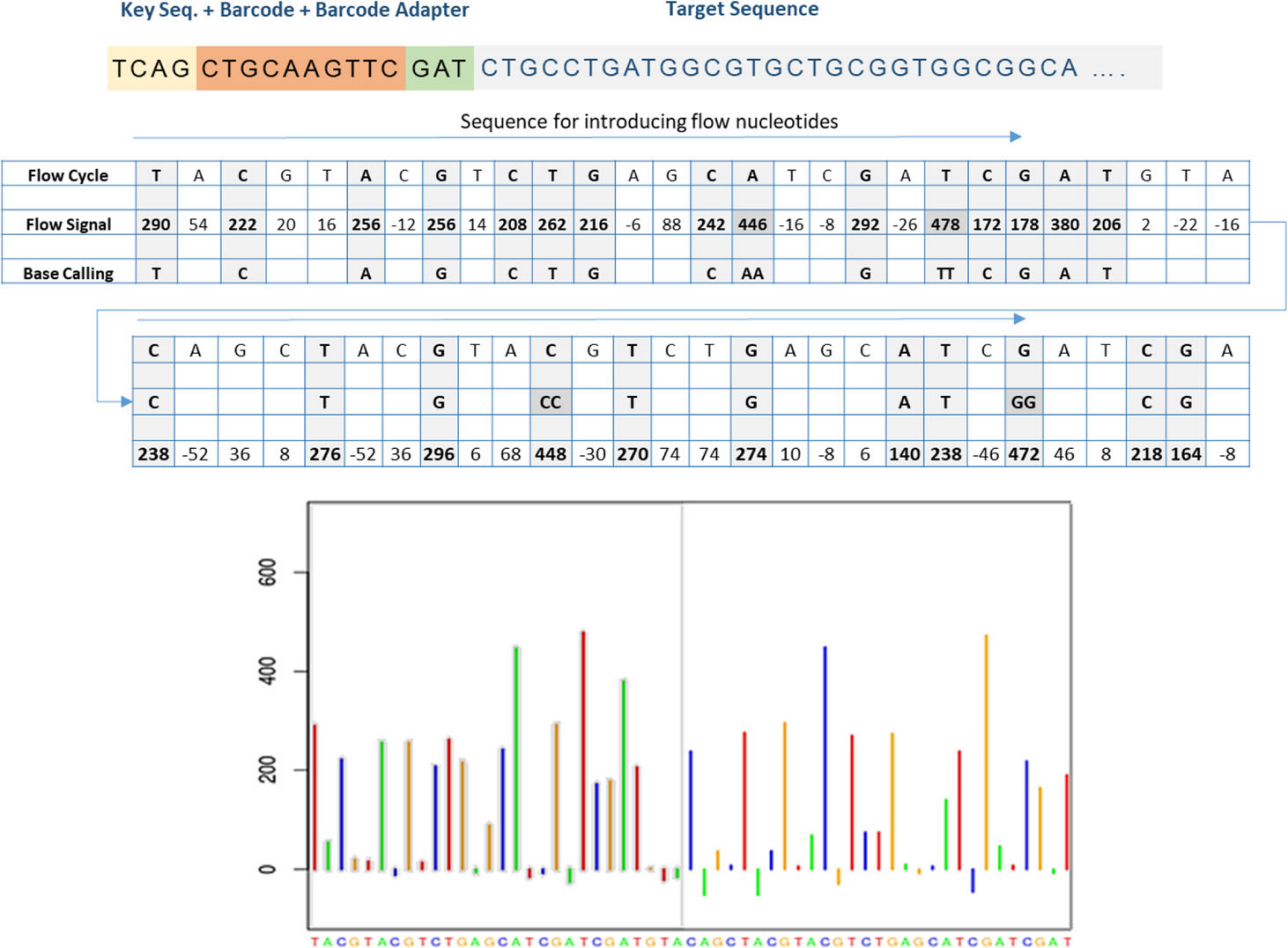
Base calling based on flow signals. The upper part shows an example DNA fragment to be sequenced. The second part shows the sequence of nucleotides in the flow cycle. It also shows the values of the sensed flow signals and the called bases. A flow signal value exceeding a certain threshold means that a base had hybridized to the template and the corresponding base in the flow cycle is reported. If the flow signal value doubles, this indicates a polymer of identical bases. The base calling software calibrates the signal values and decides the length of the polymer

Figure 2 explains the steps of the base calling by demonstrating how the flow signals are analyzed to call the bases of an example fragment using a given flow cycle. The base calling software uses the flow signals to call the bases in the target DNA as follows: The algorithm simultaneously scans the flow signal and the bases in the flow cycle. If there is a signal peak exceeding a certain threshold, then the corresponding base in the flow-cycle is the base in the target DNA and it is reported. If the flow signal value doubles, this indicates a polymer of identical bases. The base calling software calibrates the signal values and decides the length of the homopolymer. One can see in the figure that the flow signals can have ‘noisy’ negative values (around zero). Theoretically, the flow signal value can go to infinity for a DNA fragment of infinite number of the same nucleotide, (e.g., AAAAA…); but this does not occur in practice.

### The compression algorithm

Our approach to improve the compression of the Ion Torrent BAM file is based on improving the compression of the flow signals. The idea of our algorithm is that the reads with similar sequences aligned to the same locus should have similar flow signals. Therefore, exploiting such similarity across multiple identical reads would lead to better compression.

Our algorithm sorts the reads in the BAM file first by genomic coordinates then by their prefix via sorting the respective CIGAR string in order to bring the similar reads closer to each other. By scanning the sorted reads, the algorithm identifies blocks of reads mapped to the same locus. We collect the flow signals in each block and compress them together as detailed in the algorithm below. Other fields of the BAM file are compressed using Scramble [20]. The details of our method is in Algorithm *IonCRAM-CompressBAM,* presented below.

We implemented Algorithm 1 in the IonCRAM program. In actual implementation, Steps 2 and 3 are implemented together via Linux pipes. For Step 4.1, it is worth mentioning that we tried a different option to select a reference vector other than *F*_*1*_, such as computing a median vector among all *F* vectors in *B.* The other option increased the running time and did not lead to tangible improvement of compression. So, we decided to use *F*_*1*_ as the reference flow signal vector.

For Step 4.3, there are multiple options for compressing the flow signals: XZ (https://tukaani.org/xz/), gzip (http://www.gzip.org), or zstd (https://github.com/facebook/zstd). The XZ method is the default one. All these implementations are based on the dictionary based approach using Lempel Ziv decomposition. Each tool implements different tuning steps in terms of encoding and algorithm engineering. The gzip technique is based on the LZSS method which is a variation of the LZ77 algorithm as well as on Huffman coding [23]. The XZ algorithm is also based on LZ77 plus Huffman coding but is enhanced with Markov chain models to further improve the compression (The Tukaani project: https://tukaani.org/xz/). Zstd is a Facebook developed package, also based on LZ77 but enhanced with tuned levels of compression using Finite State Entropy [24]. Zstd follows speed-first design approach and it can provide ultra-compression ratios.

For Step 5, we use the Linux tar package for creating an archive of all compressed files. This archive includes the CRAM file for the input BAM file minus the flow signals computed in Steps 2 and 3, and the compressed blocks for the flow signals computed in Step 4.

The decompression algorithm starts with un-archiving the tar folder using the tar program. Then we use Scramble to decompress the CRAM part. For each block of the compressed flow signals, the *V* vectors are decompressed and the *D* vectors are reconstructed. The reference *F*_*1*_ is used to reconstruct *F*_*2*_ vector using both *D*_*1*_ and *F*_*1*_ via the equation *F*_*2*_ = *F*_*1*_ - *D*_*1*_. Then *F*_*2*_ is used to reconstruct *F*_*3*_ using the equation *F*_*3*_ = *F*_*2*_ – *D*_*2*_. The vector *F*_*3*_ is used to reconstruct *F*_*4,*_ and so on. The decompressed flow signals are finally added to the BAM file.

### Parallel processing

Parallel processing is used in IonCRAM at different levels in compressing the BAM files. First, the flow signals of the forward and reverse reads are processed in parallel. Second, the compression of the blocks to compress the flow signals can also run in parallel. Third, Scramble compresses the BAM file minus the flow signal in parallel. Finally, one can decompose the BAM file intro sub-files, each correspond to a certain genomic region. These regions are independent from one another and they can be also processed in parallel.

Parallel processing is also used during decompression. We decompress the BAM part which was compressed by Scramble in parallel with decompressing the flow signals. Also the compressed flow signal blocks are decompressed in parallel.

## Results and discussion

### The test dataset

The genome NGS file (ERR317482) used to test the original CRAM tool [19] and Scramble [20] was produced by Illumina. Later, the MPEG HTS consortium has compiled genomic test data to evaluate the available compression methods at that time [17]. The benchmarking dataset included many genomic files from different technologies and different organisms. However, there is only one small genome (ERR303541) from the early days of NGS, sequenced at very low depth of 0.6X. This low depth is no longer used in practice, neither in research nor in clinical diagnosis. To cope with recent advances in the Ion Torrent technology, we compiled a dataset for Ion Torrent BAMs, whose depth is similar to what is used in clinical practice (Table 1). This set includes four public standard exomes from the NIST and “Genome in a Bottle” Consortium [25]. These exomes were sequenced using modern Ion Proton platform with the following parameters: Ion AmpliSeq™ Exome RDY Kit for library preparation, with a mean insert size of 215 bp, Ion PI™ Sequencing 200 Kit v4 for sequencing, and Torrent Suite v4.2 for base calling and alignment. For an up-to-date version of the kits, chemistry, and analysis package, we also added a set of three test exomes and eleven test gene panels, generated at clinical grade quality from the Saudi Human Genome Program. They were sequenced using the Ion Proton Platform with the following parameters: Ion Proton Hi-Q kits for library preparation, Ion PI Hi-Q Sequencing 200 Kit for sequencing, and Torrent Suite v5.0.4 for base calling and alignment. All these files are available to download from the program website.

**Table 1:** Test datasets

Bam Name	Source	Bam Size GB	No. Reads	Average Depth (reads)
GP1.bam	In-house	1.04	1,675,346	75
GP2.bam	In-house	1.1	1,747,374	80
GP3.bam	In-house	1.46	2,355,416	109
GP4.bam	In-house	1.01	1,603,486	145
GP5.bam	In-house	1.47	2,362,387	210
GP6.bam	In-house	1.41	2,269,592	201
GP7.bam	In-house	1.44	2,352,413	470
GP8.bam	In-house	1.42	2,271,890	485
GP9.bam	In-house	2.13	3,480,496	711
GP10.bam	In-house	2.14	3,494,124	1053
GP11.bam	In-house	2.83	4,637,535	1400
WES1	In-house	53.6	91,339,566	172
WES2	In-house	60.2	97,605,030	202
WES3	In-house	57.3	92,927,166	293
HG002_NA24385_SRR1767406	NCBI (ftp://ftp-trace.ncbi.nlm.nih.gov/giab/ftp/data/ AshkenazimTrio/HG002_NA24385_son/ion_exome/HG002_NA24385_SRR1767406_IonXpress_020_rawlib_24028.bam)	50.0	82,654,309	201
HG003_NA24149_SRR1767411	NCBI (ftp://ftp-trace.ncbi.nlm.nih.gov/giab/ftp/data/ AshkenazimTrio/HG003_NA24149_father/ion_exome/HG003_NA24149_SRR1767411_IonXpress_022_rawlib_24022.bam)	43.8	73,777,136	185
HG004_NA24143_SRR1767448	NCBI (ftp://ftp-trace.ncbi.nlm.nih.gov/giab/ftp/data/ AshkenazimTrio/HG004_NA24143_mother/ion_exome/HG004_NA24143_SRR1767448_IonXpress_024_rawlib_24026.bam)	50.6	83,487,089	214
NA12878	NCBI (ftp://ftp-trace.ncbi.nlm.nih.gov/giab/ftp/data/ NA12878/ion_exome/IonXpress_020_rawlib.hg19.bam)	25.1	41,792,386	131

The table includes gene panel and exome data used for measuring the performance of IonCRAM. The BAM file size is given in MB and GB. The size of the target region is 57.7 Mbp for whole exome sequencing and about 0.48 Mbp for gene panels. The average depth is the average number of reads covering a target base.

### Measuring the flow signal content

As we mentioned in the introduction, the flow signals occupy a big portion of the BAM file. In this experiment, we measured how big that portion is in the test dataset. Also in this experiment, we measured the size of the flow signals in the corresponding CRAM files, after converting the BAM files into CRAM format using the program Scramble. Table 2 shows that the size of the flow signals in the BAM and CRAM files. The results show that the flow signals occupy about 75 and 77% of the BAM and CRAM file size, respectively.

**Table 2:** Flow signal size in the BAM and CRAM files in GB

Bam Name	Bam Size GB	Flow signal Size (GB)	% Flow signal in BAM	Flow signal size in CRAM	%Flow Signal in CRAM
GP1.bam	1.02	0.77	75.5%	0.52	77.4%
GP2.bam	1.06	0.80	75.5%	0.54	77.5%
GP3.bam	1.43	1.08	75.7%	0.73	77.6%
GP4.bam	0.98	0.74	75.9%	0.50	77.58%
GP5.bam	1.44	1.09	75.8%	0.73	77.5%
GP6.bam	1.37	1.04	75.8%	0.70	77.47%
GP7.bam	1.40	1.07	75.9%	0.71	77.62%
GP8.bam	1.38	1.06	76.4%	0.71	76.90%
GP9.bam	2.08	1.59	76.3%	1.06	77.52%
GP10.bam	2.09	1.60	76.5%	1.07	77.76%
GP11.bam	2.76	2.12	76.6%	1.41	77.67%
WES1	53.64	39.80	74.2%	26.42	76.94%
WES2	60.25	45.24	75.1%	30.11	77.47%
WES3	57.33	43.60	76.1%	29.18	78.15%
HG002_NA24385_SRR1767406	50.04	37.66	75.3%	26.73	78.03%
HG003_NA24149_SRR1767411	43.79	33.00	75.6%	23.41	78.12%
HG004_NA24143_SRR1767448	50.60	38.20	75.6%	27.09	78.15%
NA12878	25.11	18.78	74.8%	13.33	77.86%

### Measuring the space saving

In this experiment, we measured the compression power of IonCRAM compared to the BAM and CRAM formats. For the CRAM format, we used the program Scramble to compress the test BAM files in the CRAM format. Scramble is currently the most stable, optimized and popular implementation of the CRAM related methods, and its techniques are now part of the samtools/htslib package. Best compression options for Scramble were used (−9 for highest level of compression, −Z for using lzma method, and –p –P for preserving all tags in SAM/BAM file). The same options are also used when IonCRAM invokes Scramble to compress the part of the BAM file not including the flow signals (Step 2 Algorithm *IonCRAM-CompressBAM*). The experiments ran on a Dell server R940 of 88 physical Cores (Intel Gold 222), 128 GB RAM, and 8 TB SSD disks with Centos 7 OS. As a measure of compression, we use the percent space saving defined as follows:

$$ {\displaystyle \begin{array}{l} space\ saving=\left(1\hbox{-} \frac{compressed\ file\ size}{uncompressed\ file\ size}\right)=\left(1\hbox{-} \frac{1}{compression\ ratio}\right)\\ {} percent\ space\ saving=100\ast space\kern0.5em saving\\ {}\ \end{array}} $$

We used this measure because it directly reflects the amount of saving in physical storage, which directly leads to cost reduction.

Table 3 shows the results of compressing the test BAM files using Scramble and our program IonCRAM. The table also shows the results for a naïve implementation, where the flow signals are removed from the BAM file and compressed without any pre-processing. That is, in the naive implementation, no pre-processing as explained above in Algorithm *IonCRAM-CompressBAM* is performed. The table shows the average file sizes and average space saving for each group of files. Supplementary File 1 includes the details for each test file.

**Table 3:** Space saving of Scramble and IonCRAM

File	Bam Size (GB)	CRAM Size (GB)	Scramble CRAM %saving of flow signal compared to BAM	Overall Scramble CRAM %Saving w.r.t. BAM	Naïve (Scramble + XZ/Zstd)	%saving Naïve	IonCRAM Size (GB)	IonCRAM %saving of flow signal compared to BAM	Overall IonCRAM %Saving w.r.t. BAM	Improvement over CRAM
**Gene Panels**										
**Average values for Gene Panels**	1.55	1.02	33.03%	34.36%	1.28	17.11%	0.89	45.09%	42.60%	8.24%
**Min values for Gene Panels**	1.02	0.67	32.82%	34.00%	0.85	16.77%	0.59	44.73%	42.39%	7.46%
**Max values for Gene Panels**	2.76	1.82	33.23%	35.00%	2.28	17.44%	1.59	45.67%	42.90%	8.90%
**In house Exomes**										
**Average values for In house Exomes**	57.07	36.85	33.37%	35.33%	48.61	18.19%	32.17	45.36%	43.59%	8.31%
**Min**	53.64	34.34	33.06%	35%	44.51	13.89%	30.31	44.97%	43.37%	7.49%
**Max**	60.25	38.87	33.61%	36%	51.88	22.36%	34.12	45.94%	43.90%	9.06%
**Public Standard Exomes**										
**Average values for public Exomes**	42.39	29.01	29.05%	31.75%	36.57	13.71%	25.445	41.71%	39.84%	8.09%
**Min**	25.11	17.12	29.01%	31%	21.75	13.33%	14.99	41.34%	39.7%	7.80%
**Max**	50.60	34.67	29.09%	32%	43.72	14.55%	30.35	41.92%	40.0%	8.70%

Space saving of Scramble and IonCRAM for groups of test files. The second and third columns include the BAM and CRAM file sizes in GB, respectively. In Column 4, we show the saving in flow signal achieved by Scramble in the CRAM format. In Column 5, the overall space saving achieved by Scramble in the CRAM format compared to the original BAM file. Columns 6, 7, and 8 show the IonCRAM file size, percent saving in flow signals and overall saving, respectively. The final column compares overall percentage saving of IonCRAM compared to the respective CRAM files produced by Scramble.

The results show that the naïve method could achieve improvement in compression compared to the BAM file. But its performance is still inferior to Scramble and IonCRAM. The results also show that IonCRAM achieves consistent improvement by about 7.5–9% compared to Scramble. The space saving with respect to the BAM file has improved to reach a range between 40 and 44%. In other words, we use 56% of the storage space for storing an NGS file. From the experiments, we observe little improvement of compression when the depth increases. The gene panel files with higher depth are compressed little bit better than those with lower depth. Another observation is that the space saving of the four public exomes is less than that of the in-house BAM files (WES1-WES3). The reason for this is that these public exomes were sequenced using older chemistry and an older base calling program. The new chemistry achieves more consistent readings of the signal at the same position in the read and accordingly lead to more similar flow signal value, which ultimately leads to better compression.

### Testing different compression options

Our implementation of IonCRAM includes three compression options: gzip (version 1.5), XZ (version 5,2,2), and Zsdt (version 1.4.4). Table 4 shows the performance of IonCRAM using these different compression options to compress the flow signal part. To achieve maximum compression, we used the option “--ultra” for Zstd, and “-9” for gzip and XZ. It can be observed that XZ achieves the best compression. Zsdt is in second place with very comparable results to XZ. The gzip tool is in third place with a reduction in space saving of about 2%.

**Table 4:** Space saving of IonCRAM using different options

File	Bam Size (GB)	CRAM Size (GB)	IonCRAM Size with xz (GB)	IonCRAM %Saving w.r.t. BAM (xz)	IonCRAM File Size (MB, gzip)	IonCRAM %Saving (gzip)	IonCRAM size (zstd)	IonCRAM %Saving (zstd)
**Gene Panels**								
**Average values for Gene Panels**	1.55	1.02	0.89	42.60%	0.92	40.56%	0.90	42.10%
**Min**	1.02	0.67	0.59	42.46%	0.61	40.13%	0.59	41.83%
**Max**	2.76	1.82	1.59	42.90%	1.63	41.13%	1.59	42.40%
**In house Exomes**								
**Average values for In-house Exomes**	57.07	36.85	32.17	43.64%	33.80	40.77%	32.84	42.46%
**Min**	53.64	34.34	30.31	43.37%	31.84	40.53%	30.86	42.18%
**Max**	60.25	38.87	34.12	44.06%	35.83	41.14%	34.83	42.72%
**Public Standard Exomes**								
**Average values for public Exomes**	42.39	29.01	25.45	40.04%	26.46	37.59%	25.86	40.01%
**Min**	25.11	17.12	14.99	39.70%	15.64	37.42%	15.27	39.70%
**Max**	50.60	34.67	30.35	40.31%	31.56	37.74%	30.82	40.31%

Space saving of IonCRAM using different options: Columns 4, 6, and 8 show the average file sizes after compression using the options xz, gzip, and Zstd, respectively. Columns 5, 7, and 9 show the percentage space saving with the options xz, gzip, and Zstd, respectively.

### Measuring the running time and RAM consumption

**Fig. 3:**
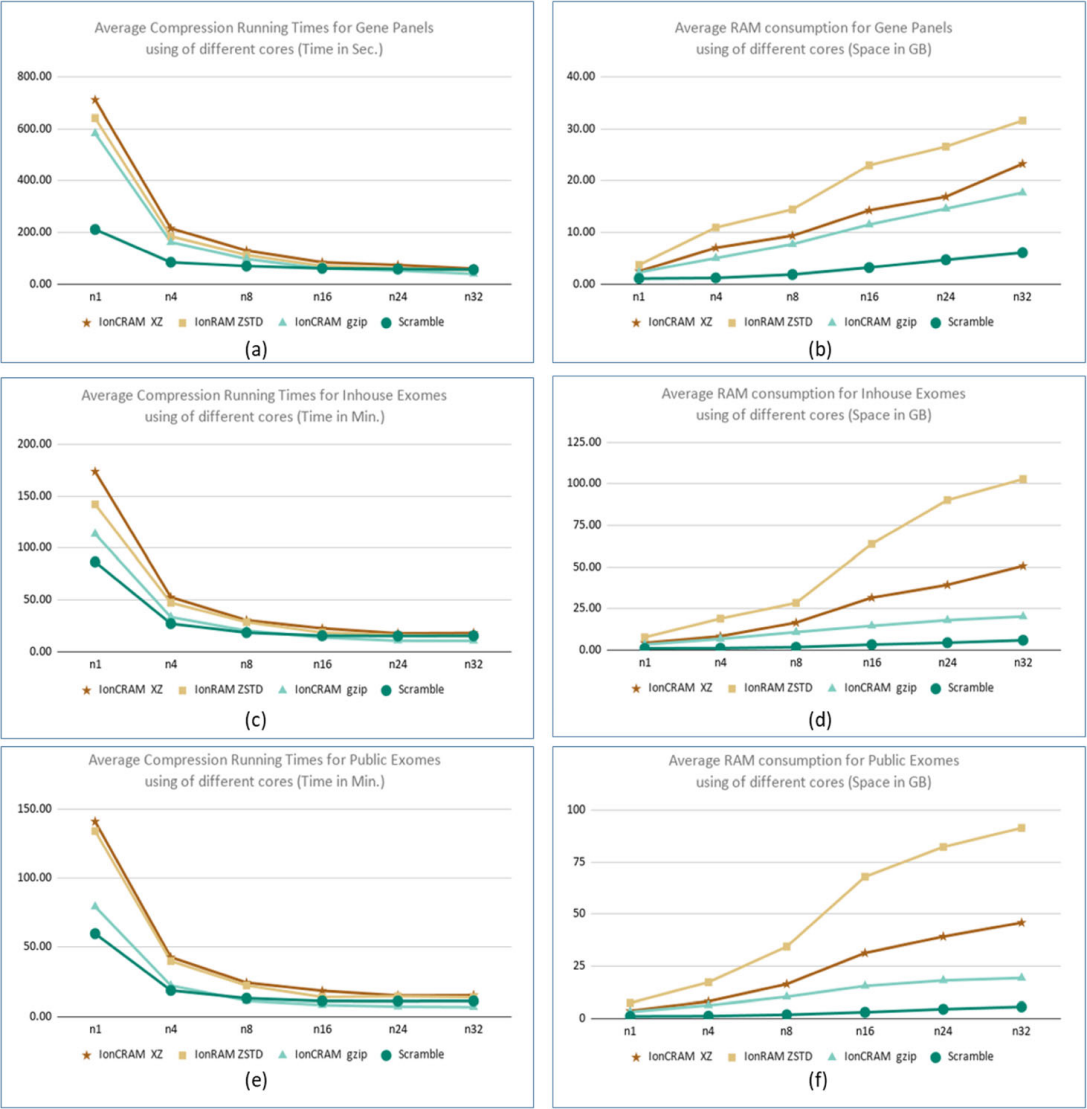
Compression Running times and space consumption. Compression running times and RAM consumption: Average running times for compressing (**a**) gene panels in seconds, (**c**) in-house exomes in minutes, (**e**) and public exomes in minutes. The measurements are for using Scramble and for using IonCRAM with the gzip, xz, and Zstd options. The average running time for gene panels is the average running times of the 11 gene panel files, and so did we for the set of the three public exomes and the set of four public exomes. The average RAM consumption in GB for gene panels, in-house exomes, and public exomes is shown in (**b**), (**d**), and (**f**)

**Fig. 4:**
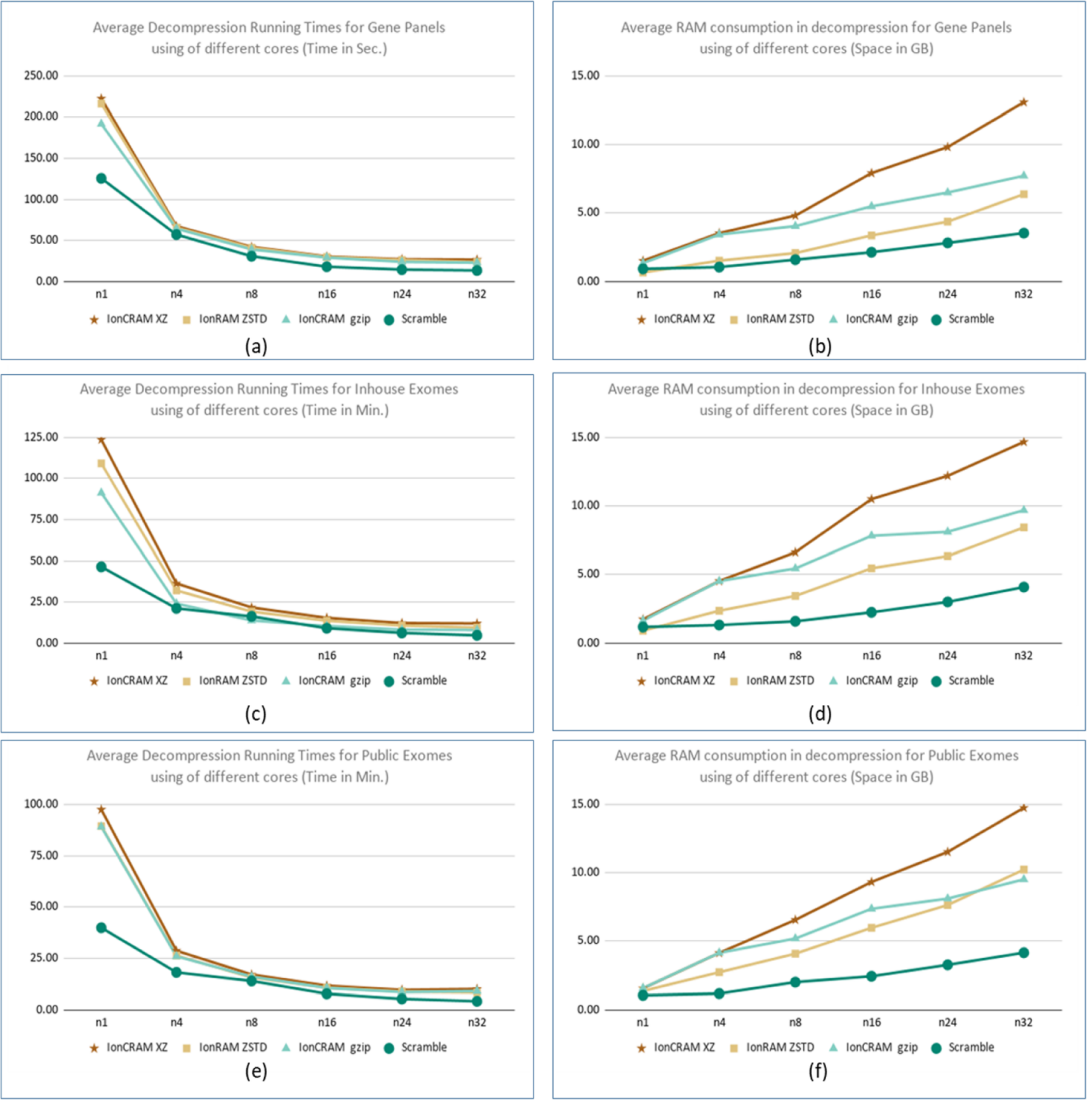
Decompression Running times and space consumption. Decompression running times and RAM consumption: Average running times for decompressing (a) gene panels in seconds, (**c**) in-house exomes in minutes, (**e**) and public exomes in minutes. The measurements are for using Scramble and for using IonCRAM with the gzip, xz, and Zstd options. The average running time for gene panels is the average running times of the 11 gene panel files, and so did we for the set of the three public exomes and the set of four public exomes. The average RAM consumption in GB for gene panels, in-house exomes, and public exomes is shown in (**b**), (**d**), and (**f**)

Figures 3 and 4 summarize the running time and RAM consumption when running Scramble and IonCRAM using different options. Supplementary File 1 includes detailed experiments in tabular and graphical formats. As expected, IonCRAM takes more time and uses more RAM than Scramble. This is mainly due to the extra work for processing the flow signals. The running time of IonCRAM improves when it runs in parallel using multiple cores, which shows very good scalability. For whole exome sequencing, it takes about 30 min in average using 8 cores and it takes 18 min using 24 cores. This is very affordable to cope with the rate of data production, even for labs with moderate computing power. (An Ion Torrent system is usually shipped with a tower server with 16 cores and 64 GB RAM.) We did not observe significant speedup beyond 24 cores. The RAM consumption increases with the use of more cores. It does not increase beyond 64 GB. From the results, we would recommend best parameters at 24 cores if high specification server is available. For workstations with moderate computing power, we would recommend the use of 16 cores so that the memory consumption does not exceed 32 GB RAM.

We also tested the running times and space consumption of IonCRAM with the gzip, xz, and Zstd options. The use of gzip option in IonCRAM leads to the best running time and best RAM consumption. It can be 50% faster and saves about 50% of the RAM consumption. Using Zstd leads to running time that is little bit faster than that of xz but still slower than gzip. As indicated by the Zstd authors in the program manual, the “ultra compression” option uses huge RAM; we could observe that in the experiment.

Recalling from Table 4 that the space saving of IonCRAM using gzip is 2% less than that using xz, then there is a kind of a trade-off, In our view, we think that the differences in running time, which is still in the range of few minutes, cannot weigh out the advantage of extra saving in storage.

### Extra experiments

#### Use of median flow signal

As explained in the Methodology section, we use the first flow signal *F*_*1*_ as a reference sequence, from which differences are computed. We also tried to use the median sequence as a reference instead of *F*_*1,*_ but the results shown in the supplementary file (Sheet 2) shows no improvement. (The median flow signal is composed of the average signal value of each point, which minimizes the total distances. Specifically, for a block of signals *F*_*1*_ .. *F*_*j,*_ the median signal *F*_*m*_ is computed as follows: *F*_*m*_[*i*] = average (*F*_*1*_[*i*] .. *F*_*m*_[*i*]), where 1 ≤ *i* ≤ *n.* The average value is the point that minimizes the function (*F*_*m*_[*i*] - *F*_*1*_[*i*]) + .. + *(F*_*m*_[*i*] - *F*_*m*_[*i*]). The reason why taking *F*_*1*_ as a reference has better performance may be attributed to better locality and is favored by the subsequent XZ and gzip compression.

#### Comparison to genozip and use of binning option

We also compared IonCRAM to the program genozip [26], using the lossless and lossy (−-optimize) options. Supplementary File 1 (Sheet 2) shows the results of this experiment. For the lossless version, IonCRAM and Scramble is superior to genozip. For the lossy version based on binning the flow signal values, genozip performs well compared to the equivalent binning option of IonCRAM. The binning option sets the negative values to zeros and maps the flow signals to certain bins, similar to the binning procedure introduced initially by Illumina to reduce the space of quality scores [27]. The binning options of IonCRAM allows the user to select the level of binning; I.e., the user select the value *x* to map each value *y* to $z=\Big(\left\lceil \frac{y}{x}\right\rceil \times x$). From the results in the supplementary file, one can see that the binning option to the nearest 10 (x = 10) could lead to extra 8–9% space saving.

## Conclusions

Compression of NGS data is important to reduce the storage requirement. It is also important to speed up the transmission of data and overcome the bandwidth issues. The research community has focused on compressing NGS data produced by Illumina platforms and no work addressed NGS data out of the Ion Torrent ones. The Ion Torrent files include extra technology-specific data fields not included in the Illumina file. This data is of large size and requires extra and should be specially addressed in compression. In this paper, we have presented the program IonCRAM for compressing the Ion Torrent BAM files. IonCRAM is the first program that could achieve significant lossless compression for such type of files. IonCRAM could achieve a space saving of about 43%, which improves upon the CRAM format by about 8–9%. This directly leads to great savings in storage, backup, and bandwidth cost.

Future research for reducing the space consumption of the Ion Torrent BAM files would include the binning of the flow signal and quality values. The idea of binning was initially introduced by Illumina [27] to reduce the space consumption of the quality values. This initiative was immediately followed by intensive research to optimize the binning procedure and address its effect on the downstream analysis, especially on the variant calling step [28–31]. We think that the binning of flow signals and quality data of Ion Torrent would also be successful, provided that the manufacturer contribute to this research. We added an option to IonCRAM for binning the flow signals, in a similar way to the binning method implemented in [26], and measured its effect on compression (Supplementary File 1). We left the step for investigating the effect of this binning on the downstream analysis to further research.

It is worth mentioning that IonCRAM has not been only used for the test data in the paper, it has also been used to compress and backup thousands of files for the Saudi Human Genome Program. IonCRAM is an open source and it is available for free along with the related test data at the tool website http://ioncram.saudigenomeproject.com.

## Supplementary information

**Additional file 1.**

## Data Availability

**Project name:** IonCRAM. **Project home page:** http://ioncram.saudigenomeproject.com, https://codeocean.com/capsule/0889064/tree/v2, https://github.com/ionCRAM/ionCRAM **Operating system(s):** Linux. **Programming language:** Python, C, C++. **Other requirements:** NA. **License:** GPL. **Any restrictions to use by non-academics:** No restrictions.

## Abbreviations

SAM: Sequence Alignment Mapping
BAM: Binary Alignment/Mapping
RAM: Random Access Memory
NGS: Next-generation sequencing
