# Correction to: IonCRAM: a reference-based compression tool for ion torrent sequence files

**DOI:** 10.1186/s12859-020-03766-1

**Published:** 2020-10-06

**Authors:** Moustafa Shokrof, Mohamed Abouelhoda

**Affiliations:** 1 Faculty of Computer Science, University of California at Davis, Davis, CA USA; 2 King Faisal Specialist Hospital and Research Center, Riyadh, Saudi Arabia; 3 Saudi Human Genome Program, King Abdulaziz City for Science and Technology (KACST), Riyadh, Saudi Arabia; 4 Systems and Biomedical Engineering Department, Faculty of Engineering, Cairo University, University Square, Giza, Egypt

## Correction to: BMC Bioinformatics (2020) 21:397 https://doi.org/10.1186/s12859-020-03726-9

Following publication of the original article [1], the authors identified a missing section in the published article. The missing section is given below.

**Algorithm IonCRAM-CompressBAM**

1. Sort the BAM file (if not sorted) by genomic coordinates. Then sort the reads starting at the same locus lexicographically via sorting their CIGAR string.
2. Separate the signals of the forward reads from those of the reverse ones and process each group independently (in parallel) using Steps 3 and 4.
3. Remove the flow signals from the BAM file and store them separately. Compress the remaining fields of the BAM file (sequence, quality, and other fields) using a reference based method (We use the program Scramble [20] for this step.)
4. Define blocks of flow signals, such that the reads in each block are mapped to the same locus. Each block *B* can be processed in parallel using the steps 4.1 to 4.3:
  4.1 Let *F*_*r*_ denote the *r*th flow-signal vector in *B* (*r* ∈ [1..*m*]), and let *F*_*r*_[*i*] ∈ $\mathbb{Z}$ denote the *i*th component of it, 1 ≤ *i* ≤ *n*. Take *F*_*1*_ as a reference vector and compute the difference vector *D*_*j*_, where D_*j*_[*i*] = *F*_*j*_[*i*] – *F*_*j*+*1*_ [*i*], 1 ≤ *i* ≤ *n*, 1 ≤ *j* < *m*.
  5.1 For each *D*_*j*_, allocate a vector *V*_*j1*_ of *n* bytes to store its values. If *D*_*j*_[*x*] > 255 for any *x*, then we write 255 in *V*_*j1*_[*x*] and write the values (*D*_*j*_[*x*]-255) in a separate list *V*_*j2*_. (The length of *V*_*j2*_ list equals the number of values larger than 255 in *D*_*j*_ and they are very rare in practice.)
  6.1 Concatenate *F*_*1*_ and the *V* vectors and compress them. (We use the XZ algorithm as default method for that purpose.) (*F*_*1*_ is a reference flow signal vector that will be used in decompression).
5. Wait until all (parallel) processes finish. Use the Linux tar package to create a compressed folder including the compressed *B* blocks files and the other compressed CRAM files computed in Step 1.

The author group has been updated above and the original article [1] has been corrected.

## References

[1] Shokrof M, Abouelhoda M (2020). IonCRAM: a reference-based compression tool for ion torrent sequence files. BMC Bioinformatics.

